# Deep Neural Network Inverse Design of Integrated Photonic Power Splitters

**DOI:** 10.1038/s41598-018-37952-2

**Published:** 2019-02-04

**Authors:** Mohammad H. Tahersima, Keisuke Kojima, Toshiaki Koike-Akino, Devesh Jha, Bingnan Wang, Chungwei Lin, Kieran Parsons

**Affiliations:** Mitsubishi Electric Research Laboratories, 201 Broadway, Cambridge, MA 02139 USA

## Abstract

Predicting physical response of an artificially structured material is of particular interest for scientific and engineering applications. Here we use deep learning to predict optical response of artificially engineered nanophotonic devices. In addition to predicting forward approximation of transmission response for any given topology, this approach allows us to inversely approximate designs for a targeted optical response. Our Deep Neural Network (DNN) could design compact (2.6 × 2.6 *μm*^2^) silicon-on-insulator (SOI)-based 1 × 2 power splitters with various target splitting ratios in a fraction of a second. This model is trained to minimize the reflection (to smaller than ~ −20 dB) while achieving maximum transmission efficiency above 90% and target splitting specifications. This approach paves the way for rapid design of integrated photonic components relying on complex nanostructures.

## Introduction

Artificially engineered subwavelength nanostructured materials can be used to control incident electromagnetic fields into specific transmitted and reflected wavefronts. Recent nanophotonic devices have used such complex structures to enable novel applications in optics, integrated photonics, sensing, and computational metamaterials in a compact and energy-efficient form^1–10^. Nevertheless, optimization of nanostructures, with enormous number of possible combination of features, using numerical simulation is computationally costly. For example, computing electromagnetic field profile via finite-difference time-domain (FDTD) methods may require long simulation time, several minutes to hours depending on the volume of the photonic device, for analyzing the optical transmission response. In order to design nanostructures achieving target transmission profile, we need to perform a large number of FDTD simulations in most meta-heuristic approaches. To resolve the issue, we previously developed an artificial intelligence integrated optimization process using neural networks (NN) that can accelerate optimization by reducing required number of numerical simulations to demonstrate how NNs can help to streamline the design process^11,12^.

Deep learning methods are representation-learning techniques obtained by composition of non-linear models that transform the representation at the previous level into a higher and slightly more abstract level in a hierarchical manner^13^. The main idea is that by cascading a large number of such transformations, very complex functions can be learnt in a data-driven fashion using deep neural networks^14^. The huge success of deep learning in modeling complex input-output relationship has attracted attention from several scientific communities such as material discovery^15^, high energy physics^16^, single molecule imaging medical diagnosis^17^, and particle physics^18^. It has received some attention in optical community and there has been several recent work on reverse modeling for design of nano-structured optical components using DNN^19–25^, as well as hardware implementation of an artificial neural network^26–30^. NNs can be used to predict the optical response of a topology (Forward Design) as well as to design a topology for a target optical response (Inverse Design).

Inverse design of photonic structures were conventionally demonstrated using adjoint sensitivity analysis^31–34^. More recently, D. Liu used a tandem NN architecture to learn non-unique electromagnetic scattering of alternating dielectric thin films with varying thickness^21^. J. Peurifoy demonstrated NNs to approximate light scattering of multilayer shell nanoparticles of SiO_2_ and TiO_2_ using a fully connected NNs with a depth of 4 layers^24^. During preparation of this paper, T. Asano provided a neural network for prediction of the quality factors in two dimensional photonic crystals^26^. Inspired by this progress, we aim to train a NN that can instantaneously design an integrated photonic power divider with a ratio specified by the user. The design space for integrated photonic devices is considerably larger than previously demonstrated optical scattering applications that call for robust deeper networks such as Deep Residual Networks (ResNet)^35^.

Integrated photonic beam splitters based on a multimode interference (MMI) have been widely used to equally divide the power into the output ports. Although an arbitrary split ratio can be applied in various applications such as signal monitoring, feedback circuits, or optical quantization^36^, the design space is hardly explored due to design complexity. Tian *et al*. demonstrated SOI-based couplers with variable splitting ratio in a 15 × 15 *μm*^2^ device footprint with 60 nm bandwidth and 80% transmission efficiency^37^. Xu *et al*. optimized positioning of squared etched pixels to achieve 80% efficiency for arbitrary ratio power dividers in 3.6 × 3.6 *μm*^2^ device foot print^38^.

To design photonic power divider with arbitrary splitting ratio, the designer often begins with an overall structure based on analytical models and fine tune the structure using parameter sweep in numerical simulations. Here, we demonstrate that using deep learning methods we could efficiently learn the design space of a broadband integrated photonic power divider in a compact deep residual neural network model. This method allows design by specifications, where user simply asks for a specific power splitting performance and can see the near ideal solution almost instantaneously without depending on time-consuming FDTD simulations. Our device has above 90% transmission efficiency in a footprint of 2.6 × 2.6 *μm*^2^, which to the best of our knowledge, is the smallest arbitrary ratio beam splitter to date. Moreover, our design does not rely on arbitrary device morphologies and is constrained to a 20 × 20 vector of etched holes with a radius of 45 nm, conveniently fabricable by the current semiconductor technology.

## Deep Learning for Forward Modeling to Predict Optical Response

### Simulation Setting and Dataset

**Figure 1:**
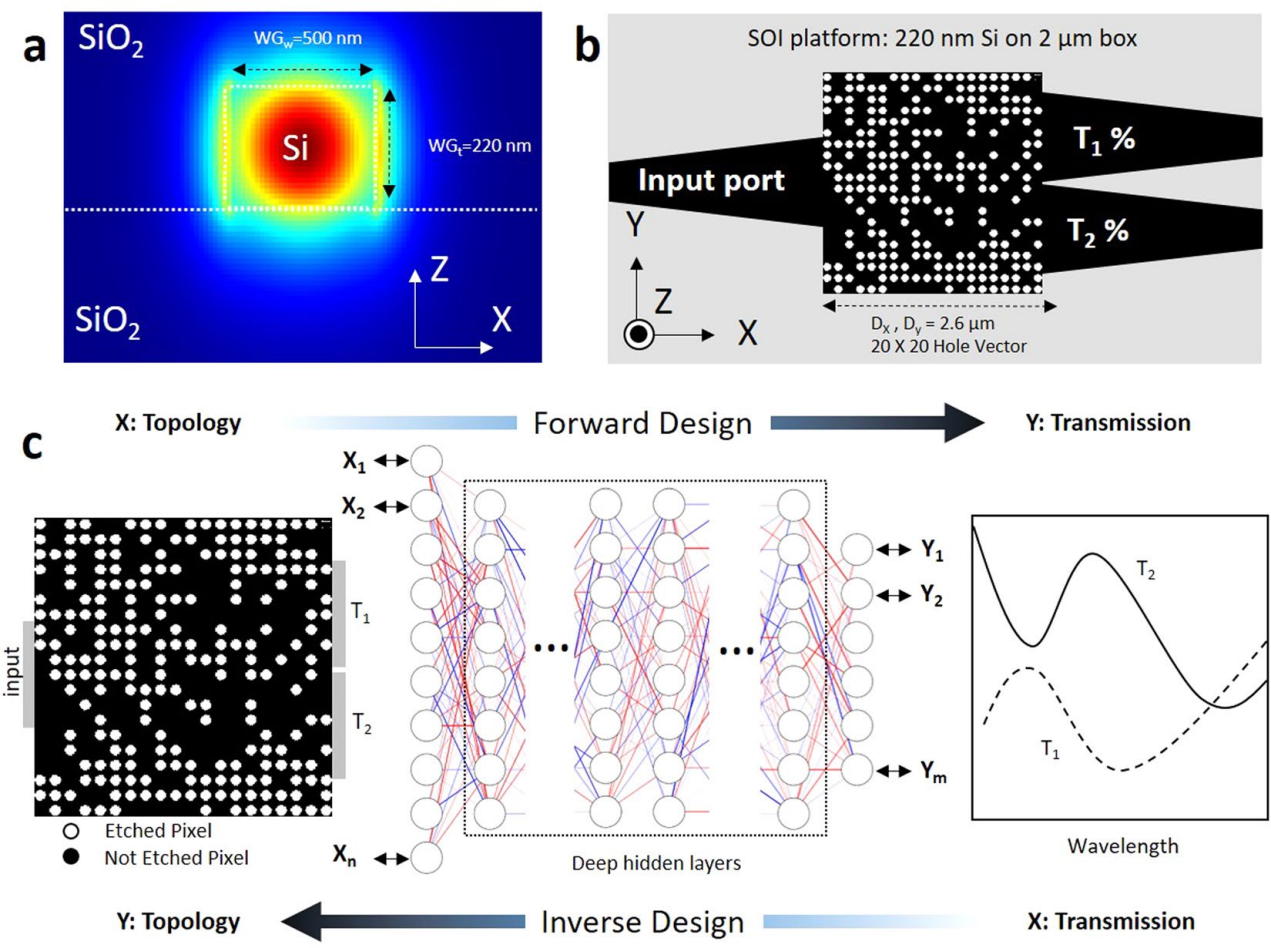
Overview of the DNN prediction and inverse design process. (**a**) TE mode is launched into the standard SOI waveguide at the input port of the power splitter (Note that the scales in x- and y-directions are different). (**b**) Schematic of a nanostructured integrated photonics power splitter with a footprint of 2.6 × 2.6 *μm*^2^. Circles indicates location of etch holes; by optimizing binary sequence of position of etch holes it is possible to adjust light propagation into either of the ports. (**c**) We use DNN for forward and inverse modeling of nanophotonic devices. The DNN can take device topology design as input and spectral response of the metadevice as label or vice versa.

When a broadband light encounters an obstacle with a different refractive index, along its path, it undergoes reflection, refraction, and scattering. The goal of the nanostructured integrated photonics power splitter is to organize the optical interaction events, such that the overall effect of the ensemble of scattering evets guides the beam to a target port and power intensity. To design the power ratio splitter using DNN we chose a simple three port structure on a standard fully etched SOI platform. One input and two outputs 0.5 *μm* wide port are connected using an adiabatic taper to the 2.6 *μm* wide square power splitter design region with a connection width of 1.3 *μm* (Fig. 1). We use numerical simulation (Methods section) to generate labeled data for training the network. We then feed the DNN with numerical optical experiments and train a neural network able to represent the relationship between hole vectors and spectral response at each port. Initially our input data are several 20 × 20 hole vectors (HV), each labeled by its spectral transmission response (SPEC) at port 1 (T_1_) and port 2 (T_2_) and reflection from the input port (R). Each pixel is a circle with a radius of 45 nm that is easily fabricable using conventional lithography methods^32,39^. Each pixel can have a binary state of 1 for etched (n = n_silicon_) and 0 for not etched (n = n_silica_) (See Methods). Changing the refractive index at a hole position modifies the local effective index inside of the power divider to determine the propagation path for the travelling wave in the device.

**Figure 2:**
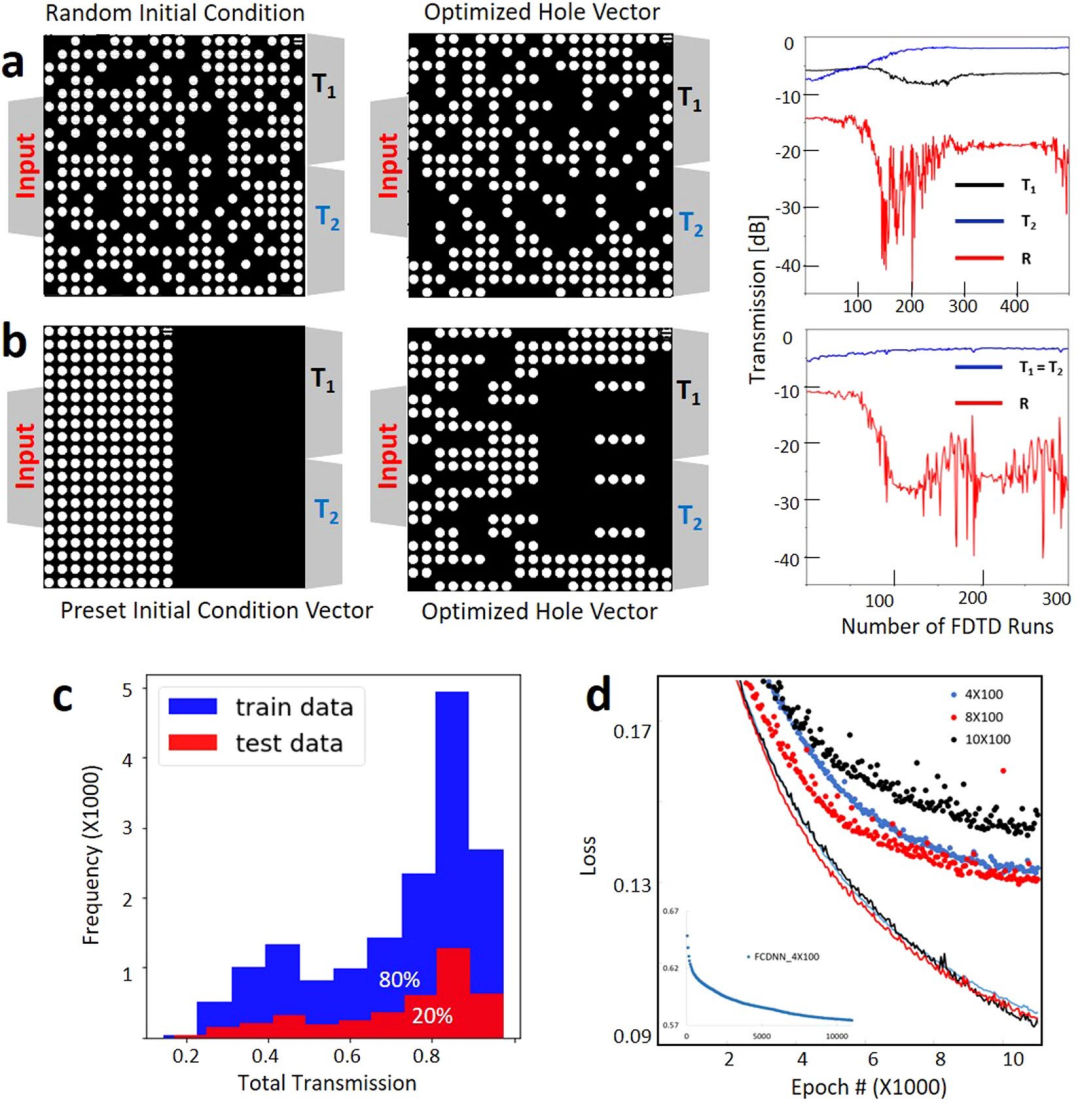
We train the DNN network with a diverse set of data. Each data set starts with an initial condition, etched hole density, and a metric to optimize a spectral response. We generate approximately ~20,000 etched hole vector as data, each associated with its transmission response as label. Here we show two of these data sets: (**a**) an asymmetric optimization search to maximize $\min ({T}_{1})+\,\min ({T}_{2})-\alpha \times \,{\rm{\max }}(|R|)\,(\alpha =2)$ with a random initial vector, (**b**) a symmetric search to maximize $\min ({T}_{1})+\,\min ({T}_{2})-\alpha \times \,{\rm{\max }}(|R|)\,(\alpha =4)$ with a patterned initial vector. T_1_ and T_2_ are transmitted power at port 1 and 2, respectively; R is the reflected optical power to the input port. For symmetric cases, the transmission for port 1 and port 2 are identical (T_1_ = T_2_). (**c**) Histogram of all transmission train and test data labels collected by numerical methods for ~20,000 power splitter topologies at 1550 nm. d) Learning curve for ~10,000 epochs of training for both training (lines) and test (dots) losses, for networks with constant hidden layer width of 100 and depth of 4, 8, and 10. Learning curve for deep residual neural network shows the network loss reduces by increasing depth of network up to 8 layers. The inset shows the best case (4 layer) for a FCDNN that has significantly larger loss value ~0.58. Here all cost functions are based on negative log likelihood.

We use randomly chosen HVs and carefully chosen patterned initial HVs (see Fig. 2a and Supplementary Fig. S1), and optimize spectral transmission values using heuristic optimization approaches for various optimization metrics to collect a diverse set of labeled training data for supervised learning. In the case of a symmetric search, we enforce symmetry of HV across the X axis and take advantage of the symmetry of the topology to reduce the search space from 400 pixels to 200. In addition, this reduces the simulation time to half because of the symmetric boundary condition. Therefore, the spectral response T_1_ and T_2_ are equal for symmetric devices (Fig. 2b). The input and output waveguide and other geometrical details of symmetric and asymmetric cases are identical. For both cases shown in Fig. 2, we start the optimization with an initial hole vector and use a single stride binary search to maximize ${\rm{FOM}}=\,{\rm{\min }}({T}_{1})+\,\min ({T}_{2})-\alpha \times \,{\rm{\max }}(|R|)$ for α = 2 and 4 for Fig. 2a and b, respectively. Our design goal is to achieve compact nanostructured power splitters with high transmission efficiency and minimized back reflection. Low back reflection is of great importance since in active integrated photonic circuits, it is important to minimize the back reflection. That is why we use reflection factors larger than 2 in our optimization metrics to emphasize minimizing back reflection in these power splitters. We repeat this process for ~ 20 different initial conditions and splitting ratio targets and add mirrored data for asymmetric cases to collect a total of ~ 20,000 data with their corresponding spectral labels (Fig. 2c).

For the forward problem, inputs are two-dimensional 20 × 20 HV arrays corresponding to binary images of hole locations. We train a DNN to predict the SPEC vector which is a one dimensional vector with 63 elements. SPEC includes broadband spectral data (1450 to 1650 nm) for transmission at output ports T_1_ and T_2_, as well as the reflection to the input port R. For the inverse design, SPEC is used as an input and hole vectors are considered as labels. The forward problem is solved as a regression problem and we use a Gaussian log-likelihood function to train the model. In contrast, the inverse problem is solved as a classification problem, where we predict the binary vectors that represent the hole locations. Therefore, we use a Bernoulli log-likelihood classifier as the loss function for training the inverse problem. The Gaussian log-likelihood loss function is represented by the following equation.

1 $$-\mathrm{log}\,P(Y|X,{\boldsymbol{W}})=\frac{1}{K}\sum _{n}^{K}(\frac{1}{2}\,\mathrm{log}(2\pi {\sigma }^{2})+\frac{1}{2{\sigma }^{2}}{({y}_{n}-{{\boldsymbol{W}}}^{T}{x}_{n})}^{2}),$$

where P(Y|X,**W**) denotes the probabilistic model, **W** denotes the model parameters, K is the number of training data. The loss function is optimized using the Adam optimization algorithm^40^. Training is terminated after a fixed number of iterations to ensure convergence (Fig. 2d). The training and validation results were similar for our trained networks and thus we didn’t use any regularization for over-fitting.

**Figure 3:**
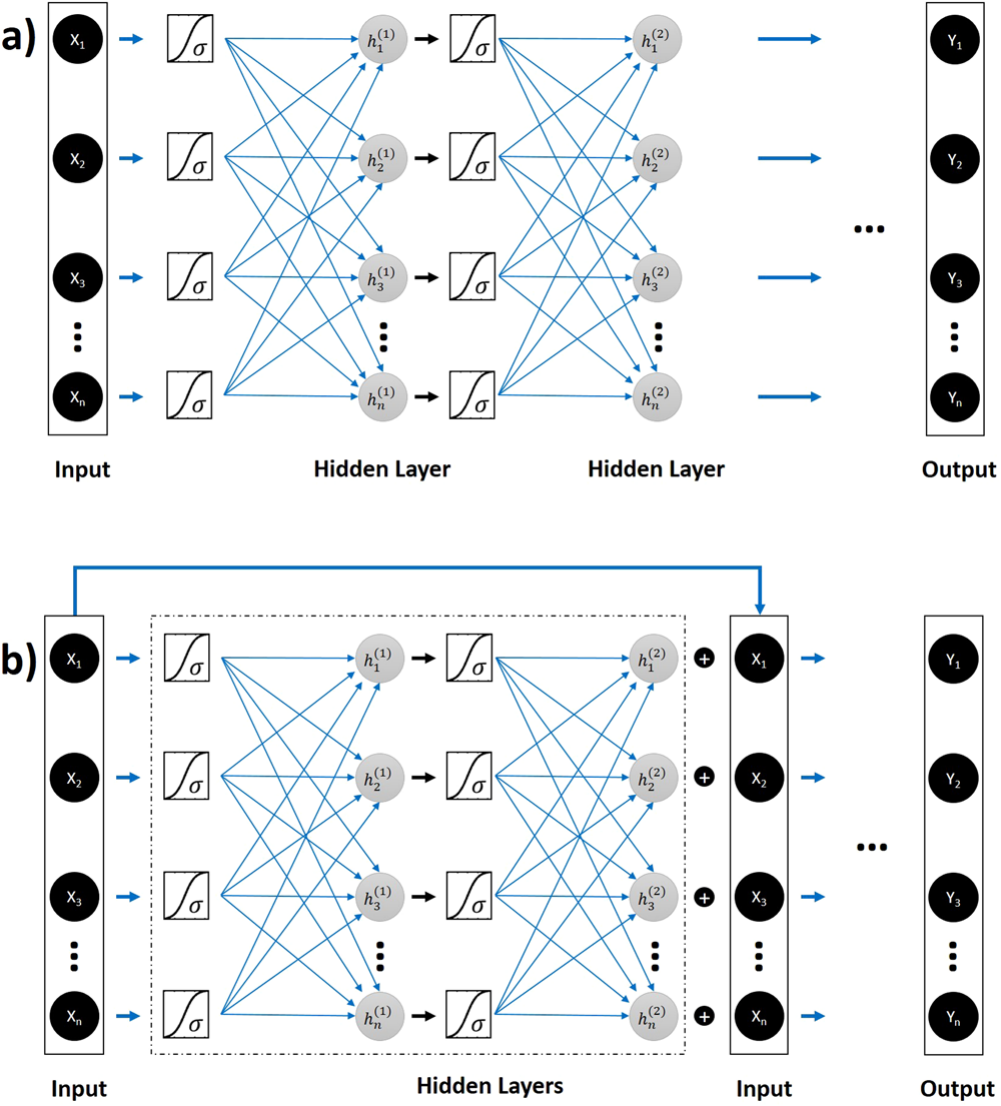
Network architectures for a plain FCDNN (**a**) and a ResNet DNN (**b**) used for the inverse design of integrated nanophotonics. We use sigmoid (*σ*) activation function in both network architectures. Increasing depth of a FCDNN doesn’t necessarily improve the performance. The ResNet is designed to use an additional identity function to achieve accuracy from increase in network depth.

For both the problems, we first used a fully-connected DNN (FCDNN) with multiple layers where each layer has 100 neurons. The number of layers was considered as a hyperparameter which was optimized during the numerical experiments. However, we found that increasing the depth of the FCDNNs didn’t improve the performance of the network. Consequently, we used a residual deep neural network (ResNet) to improve the depth of training up to 8 hidden layers for both the forward and inverse problem (see Fig. 2d for a quantitative comparison between regular DNNs and ResNets). To explain, FCDNNs generally suffer from the problem of vanishing gradients. As a result, increasing depth of a FCDNN doesn’t necessarily improve the performance. The ResNet is designed to circumvent this problem by using “identity shortcut connections” with the underlying hypothesis that it is easier to optimize the residual mapping than to optimize the original, unreferenced mapping (Fig. 3). ResNets have empirically been proven to allow more flexibility in training deep architectures than the FCDNN^35^. The main idea is that the ResNet uses an additional identity function to allow smooth forward and backward propagation of gradients.

## Results

To test the nanostructured power divider network, first we use a randomly selected, unseen 20% data from the same set of simulation data used to train the network. The test data set helps to prevent overfitting the model to the training data (Fig. 2d).

**Figure 4:**
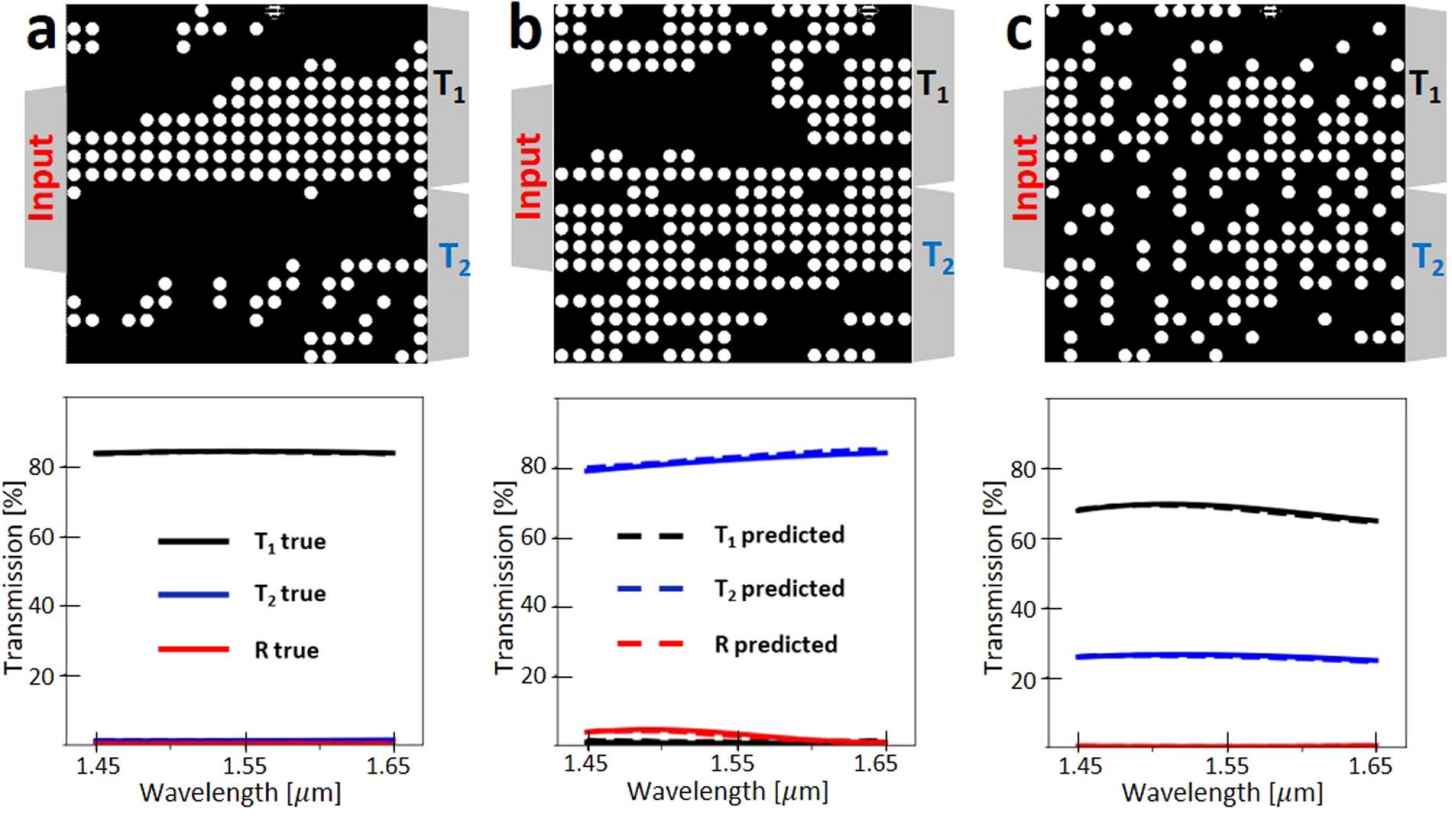
Spectrum approximation using deep ResNet. We use 16,000 (80% of the total) input data for training and 4,000 (20% of the total) data for testing. a, b, and c are comparison of ResNet predicted spectral response of the three representative power splitters to the numerically verified spectral responses. Black, blue, and red colors stand for transmission at port 1, transmission at port 2, and reflection at the input port, respectively. Solid lines are true values for a given hole vector and dashed lines are predicted spectral response using ResNet.

**Figure 5:**
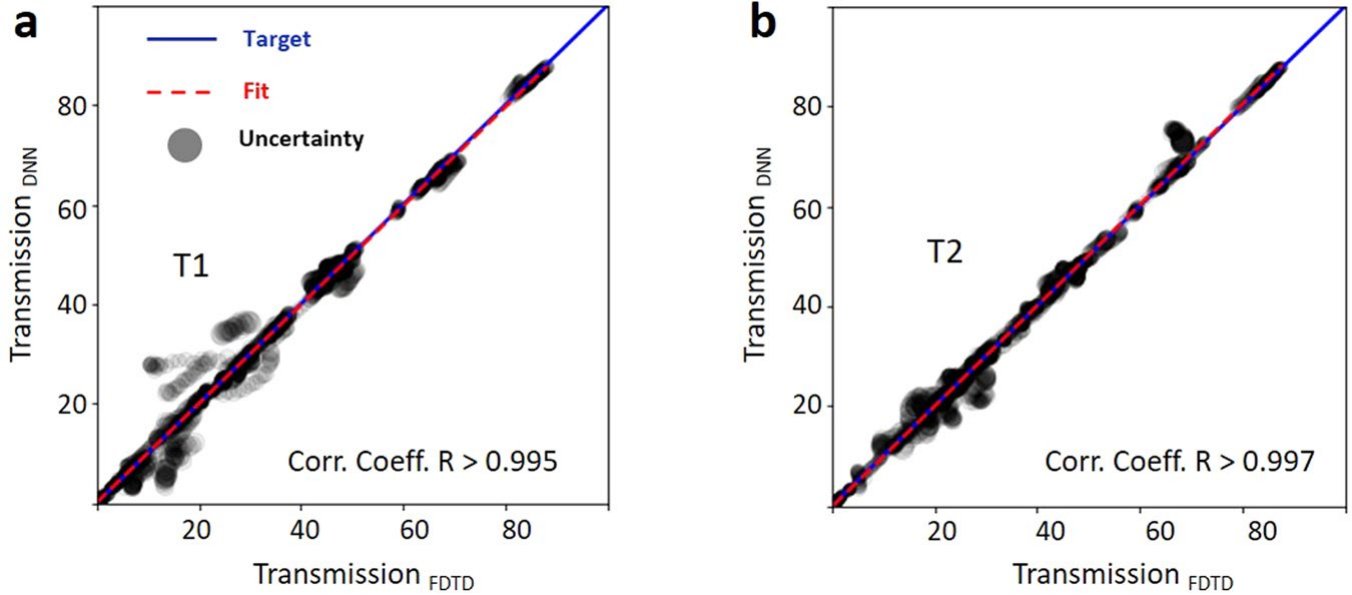
Correlation coefficient. Fitting ResNet predicted transmission values versus true transmission values for port 1 (**a**) and port 2 (**b**). The correlation coefficient R is above 0.995 across the full range of transmission ratios (0 to 1) and approaches unity as transmission increases. Gray circle symbol size is proportional to gradient uncertainty.

In the following we present the outcome of the network for forward prediction of spectra from HV (Fig. 4), and inversely designing the HV from a given physically feasible SPEC specifications (Fig. 5). First we test the forward computation of the network to see prediction of spectral response of a topology that the network is not trained on. Interestingly, the network could predict transmission and reflection spectra quite accurately (Figs 4 and 5).

To quantify the prediction accuracy we use a correlation plot that compares true numerically verified optical response with DNN predictions. The correlation coefficient of the DNN prediction was above 99% (Fig. 5). We use the variance of the negative log likelihood cost function as a means to determine the confidence of the neural network and show it as the area of the prediction uncertainty in the correlation plot. We observe that confidence of the prediction is lower in lower transmission regime and improves at higher transmission regimes. This is expected since the training data mainly contains high transmission devices (Fig. 2d).

**Figure 6:**
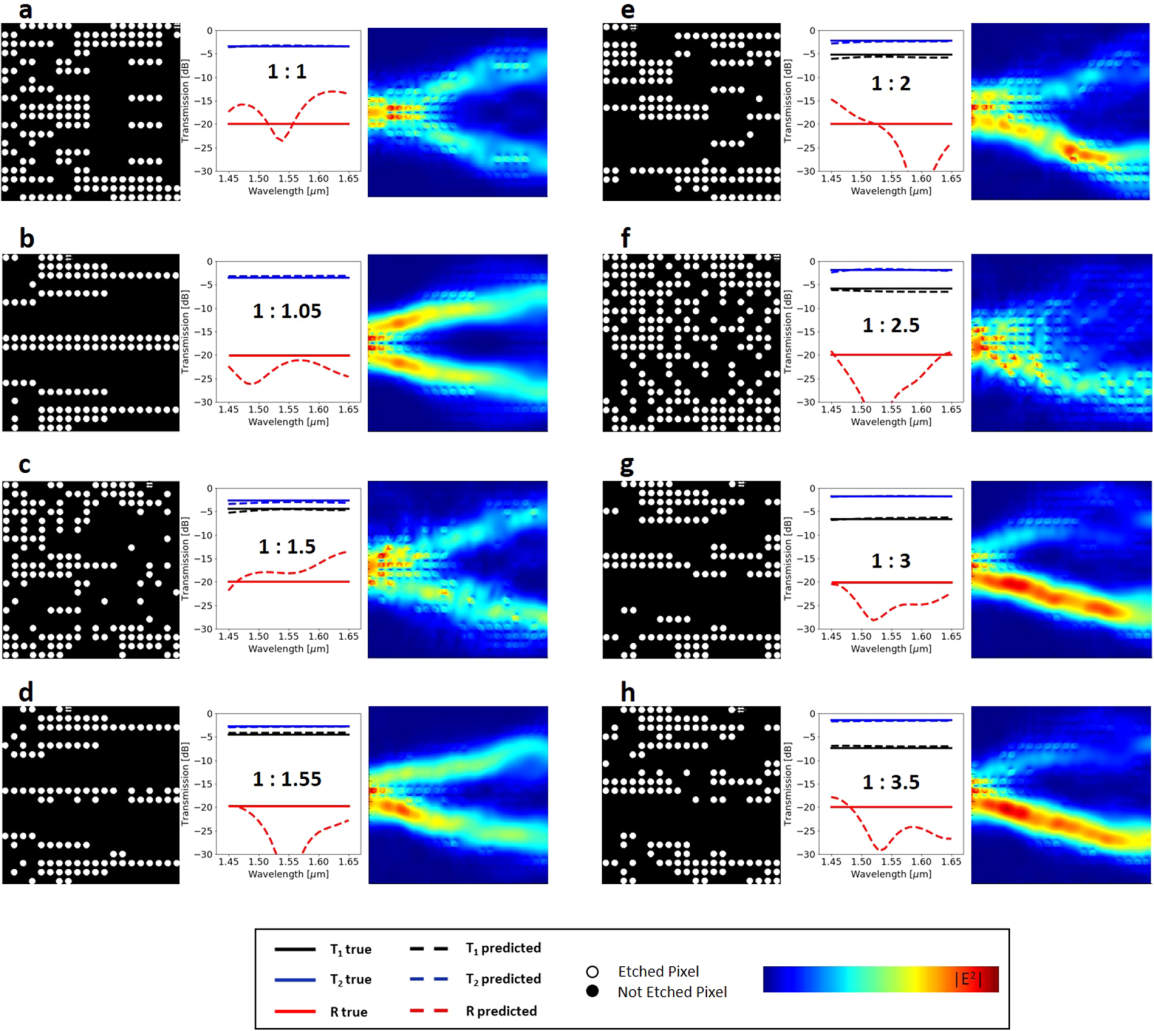
Demonstration of DNN inverse design. We use ResNet DNN inverse design for 90% efficient power splitters. Here we show 8 representative devices with splitting ratios of 1:1, 1:1.05, 1:1.5, 1:1.55, 1:2, 1:2.5, 1:3, and 1:3.5 from a to h, respectively. Spectral response plots show that the numerically verified transmitted and reflected optical powers (dashed lines) of the predicted binary patterns match well with the target broadband spectra (solid lines). Electromagnetic energy density plots (right) of each device operating at 1,550 nm are calculated using FDTD simulations.

We test the inverse modeling on the same data as above by using SPECs as data and HVs as label and reversing and optimizing the inverse network. To test the generalization capabilities of the network, we investigate the network’s inverse design performance on arbitrary and unfamiliar cases. To do this, we generate a reference table containing broadband constant transmission values for each port and use them as the input data batch for the Inverse Design DNN model. The predicted HVs can take any value from 0 to 1 from a Bernoulli distribution classifier. The classification converges to 0 or 1 as the loss reduces by increasing the number of training epochs. The produced quantized binary sequences contain features that the model is not trained on (Supplementary Fig. 2), which are then fed back into the numerical solver to evaluate the prediction performance. In a next step, we run independent FDTD simulation to check validity of the response (Fig. 6). Numerically simulated electric field propagation at center wavelength of 1550 nm for 8 splitting ratios of 1:1, 1:1.05, 1:1.5, 1:1.55, 1:2, 1:2.5, 1:3, and 1:3.5 show various power splitting mechanisms from classical MMI based beam splitters. The electric field distribution intensity in the case of 1:1 and 1:05 splitting are almost symmetric. On the other hand the electric field intensity is asymmetric for asymmetric splitting ratio devices (as expected) and beam path is broadened for the side with a larger output T_2_.

Predicted topologies for target spectral responses for a-b and c-d pairs show different looking patterns for similar spectral response. This makes sense, because there could be several solutions to a single electromagnetic problem. FDTD simulated optical power propagation through predicted power splitter are demonstrated for each pattern. In all of the eight cases shown in Fig. 6 the transmission efficiency exceeds 90% which, to the best of our knowledge, is the highest transmission efficiency demonstrated in integrated power splitters. And this is also the first time minimizing reflection is taken into consideration. Although we did not aim to maximize the operation bandwidth as an objective, our power splitters show broadband transmission between 1450 to 1650 nm. Additionally, we set the reflection target to −20 dB at 1550 nm wavelength. We achieve reflection responses less than −20 dB at 1550 nm, except for the case of Fig. 6c. The main reason for apparently large variations in predicted and actual reflection response shown Fig. 6 is because the spectral response are shown in logarithmic scale. In reality the differences are very small.

## Discussion

NNs can be used to take device structure data (shape, depth, and permittivity) to predict the optical response of the nanostructure (forward network). In this case NN can be used as method for fast approximation of the optical response instead use of computationally heavy numerical methods. Another way to use NNs, which is not available in conventional numerical methods, is taking an optical response as input and providing user with an approximate solution nanostructure (inverse design). Although DNN initially need a large amount of data set for the training purpose, it is possible to process several heuristic optimization metrics in parallel on a computing cluster to speed up generating the training data. Once the network is trained to represent the topology as optical response and vice versa, it can design the nanostructured geometry in a fraction of second.

We utilized a ResNet DNN architecture to use an additional identity function to allow smooth forward and backward propagation of gradients. This allowed us to increase the depth of the network to 8 layers. We observed that there is still some overfitting in the ResNet DNN for 10 × 100 structure (10 layer deep and 100 neuron wide). As the number of parameters learned from data depend on the number of neurons in the DNN, the 10 × 100 has the highest number of parameters learned during training. While this provides good performance during training, it leads to some overfitting of the data. It is possible to include dropout in the DNN to allow for regularization and increasing the hidden layer depth further; however, this is left as a future work.

In conclusion, we have demonstrated application of DNNs in design of nanostructured integrated photonic components. Although the design space for this problem is very large (2^400^) possible combinations), by training DNN with nearly 20,000 simulation data we trained a network that can approximate the spectral response of an arbitrary hole vector within this design space. In addition, we could use the inverse network to design a nearly optimized power splitter topology for any user specific power splitting ratio. The capability of DNN in predicting optical response of a topology and the inverse design holds promise in wide spread use of these networks in the design of nanostructured photonic systems.

## Methods

### Numerical Simulations

We use Lumerical’s FDTD simulation package to generate the training data. The data contains more than 20,000 numerical simulations, where each experiment is a 3D FDTD simulation composed of passive SOI waveguides and the beam splitter device. Initial random hole position matrix of the beam splitter was generated, exported, and manipulated using the MATLAB automation. Hole position generating script uses different algorithms (such as Direct Binary Search), initial conditions, and optimization metrics to sequentially create sufficiently large data set required for a robust neural network representation of the device structure. It took a desktop computer with a core-i7 CPU with 3.7 GHz clock speed and 64 GB RAM about two weeks to complete collecting the 20,000 simulation data.

Dispersive refractive indices of silicon and silica from literature^41^ were used for all simulations for a broadband simulation in the range of 1.45–1.65 *μm*. The fundamental TE mode at 1550 nm was used at input source and TE mode output power was recorded for transmission and reflection. We note that TM mode output is below 10^−5^.

### Deep Neural Network (DNN)

We use the open source machine learning framework of Tensorflow in python language to build and test our deep neural networks. The runtime to train the neural network model depends on network and training parameters including data size, hidden layer depth and width, batch size, and epoch numbers. The runtime to train the neural network model depends on network and training parameters including data size, hidden layer depth and width, batch size, and epoch numbers. For the representative network parameters, with hidden layer width of 100, hidden layer depth of 8, batch size of 100, epoch number of 10,000, and trained on 20,000 data, shown in Fig. 2d, it take 1337 seconds (~22 minutes) to train the model.

## Supplementary information

Deep Neural Network Inverse Design of Integrated Photonic Power Splitters
