# Supplementary material for: Deep Neural Network Inverse Design of Integrated Photonic Power Splitters
Source: Sci Rep. 2019 Feb 4;9:1368. doi: 10.1038/s41598-018-37952-2 (PMC6361971; doi:10.1038/s41598-018-37952-2)
Supplement: Supplementary file 1 — Deep Neural Network Inverse Design of Integrated Photonic Power Splitters [file 41598_2018_37952_MOESM1_ESM.pdf]

Deep Neural Network Inverse Design of Integrated Photonic Power Splitters

(Supporting Figures)

Mohammad H. Tahersima[1], Keisuke Kojima*[1], Toshiaki Koike-Akino[1], Devesh Jha[1], Bingnan Wang[1], Chungwei Lin[1], Kieran Parsons[1]

Mitsubishi Electric Research Laboratories, 201 Broadway, Cambridge, MA 02139, USA

Correspondence to: kojima@merl.com

November 12, 2018

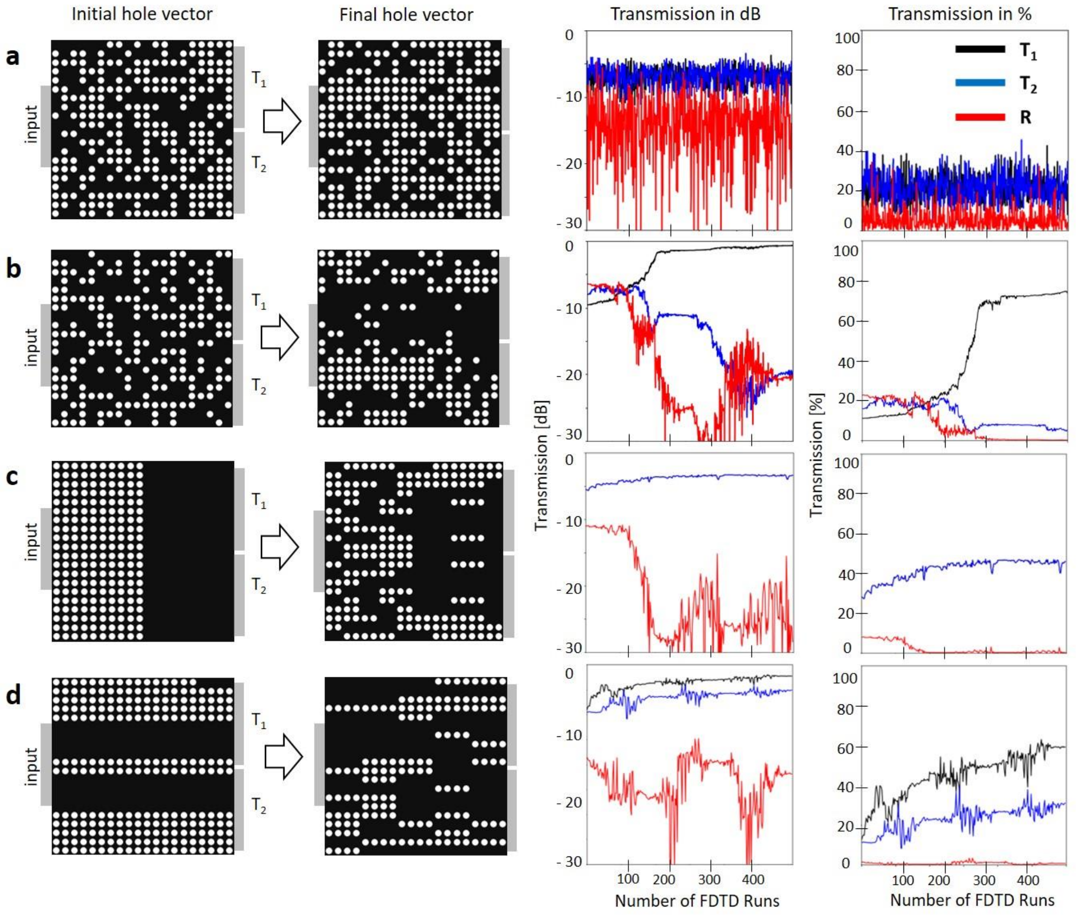

Figure S1: We train the network with a diverse set of data, each starting with an initial condition, etched hole density, and various metrics to optimize a spectral response. We generate approximately ~20,000 etched hole vector data with transmission labels. Here we show four set of these data:

a) random positioning of etch holes in the beam splitter box
b) asymmetric search optimization to maximize T_1 with an random initial vector
c) symmetric search to maximize $\min(T_1)+\min(T_2)-4*\max(\text{abs}(R))$ with a patterned initial vector
d) asymmetric search to maximize $\min(T_1) + \min(T_2) - \text{abs}(\min(T_1) - 2 * \min(T_2))$, with a patterned initial hole vector

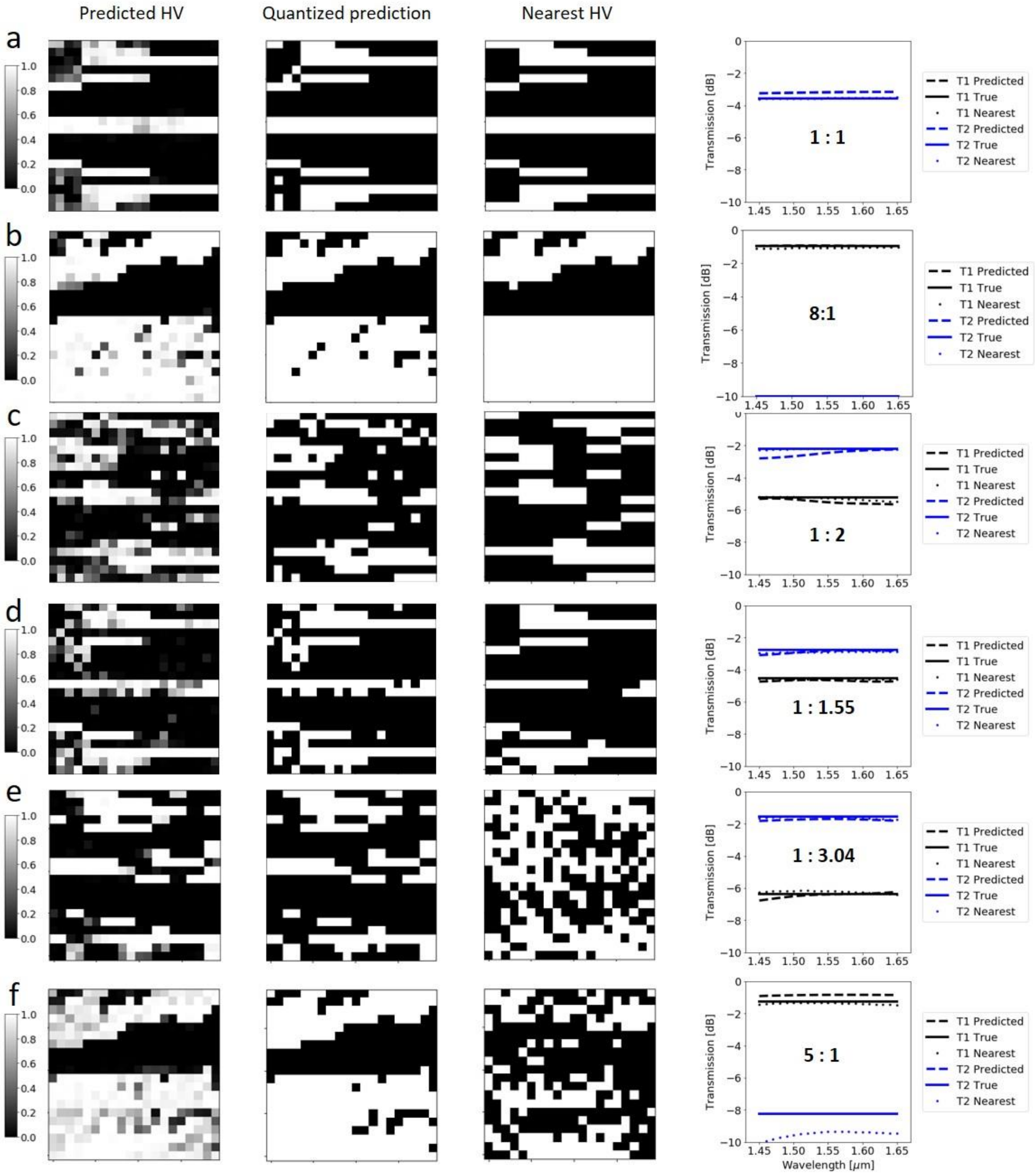

Figure S2: Comparing predicted patterns and training patterns (HV) with similar splitting ratios (SPEC) for 6 different splitting ratio. Elements of predicted hole vector can have a number between 0 and 1 (Predicted HV). We quantize this vector by rounding numbers larger than the middle point to 1 and numbers smaller than the middle point to 0 (Quantized prediction) and return it to numerical solver to calculate the spectral response of the patterned power splitter. Next, we find the nearest spectral response to the true response (desired response) and show its corresponding HV (Nearest HV). These results show that the trained model is not memorizing the training data.
